# A Pavement Piezoelectric Energy Harvester for Small Input Displacements

**DOI:** 10.3390/mi14020292

**Published:** 2023-01-22

**Authors:** Bin Yin, Jiaming Wei, Xin Jiang, Yan Liu

**Affiliations:** Key Laboratory of Electronic Equipment Structure Design, Ministry of Education, Xidian University, Xi’an 710071, China

**Keywords:** piezoelectric energy harvester, pavement, small displacement, axially deformed beam

## Abstract

In order to collect mechanical energy from human motions on pavement without an obvious disturbance, a piezoelectric harvester for small displacement is proposed. A seesaw mechanism is utilized to transmit the pressure displacement to piezoelectric beams. Benefitting from the superiority of used axially deformed beams, the designed scheme can produce a higher voltage than the ones based on the conventional bending cantilever. Favorable electrical energy is achieved by the manufactured prototype under a displacement lower than 1 mm. Two practical applications, including charging a capacitor and powering an environmental sensing node, demonstrate the feasibility of this energy harvester in supplying power for engineering devices. The proposed device shows a favorable capacity to capture energy from humans walking on pavements. Also, this category of axially deformed beam could provide ideas for developing piezoelectric harvesters under small displacements.

## 1. Introduction

With the accelerated development of city roads, intelligent traffic facilities, such as lights, road signs, and flow monitoring systems, are becoming more and more extensive [1,2]. This means that more energy is needed to maintain the continuous operation of these devices. Meantime, the motions of vehicles and humans can transmit abundant energy to the roadway, which is often dissipated into surroundings as heat and deformation. In the past decades, great efforts have been devoted to developing harvesters to collect these energies and convert them into electricity [3]. An energy harvester can be constructed by combining a mechanical structure with an energy transducer. The former commits itself to capture the mechanical energies and regularizing them into internal strain energy or kinetic energy of relevant components. Then, the transducer converts regularized energies into electricity to power low-consumption devices.

Deformation or force will act upon the surface of pavement when peoples walk through the road, and the contained potential energy can be collected by using electromagnetic or piezoelectric harvesters [4,5,6,7,8]. As a simple electromagnetic mode, the vertical displacement exerted by pedestrians can be directly loaded onto the harvester to produce a relative motion between the magnet and coil, which can generate electric currents in the coil as a linear generator according to Faraday’s principle [9]. A large displacement is required to sufficiently capture the loaded energy when only one coil-magnet pair is used, but the obvious sink due to energy harvesting will cause a great inconvenience to pedestrians. When utilizing multiple coil-magnet sets to promote the output power, a larger volume and higher cost will put extra barriers on the application path. Meantime, several mechanical systems, such as a rack-pinion and cam-arm, are used to convert the vertical displacement into rotational motion to actuate an electromagnetic generator [10,11]. Moreover, a flywheel is often installed in these systems and acts as an energy accumulator to absorb the fluctuant input energies and release them in a stable way [12]. However, the required high vertical displacement, inherent large volume, and high cost originating from multiple mechanical components still create obstacles to the wide application of electromagnetic harvesters. Piezoelectric materials have been widely used to harvest energy from mechanical vibrations, air flows, and circular/linear displacements [3,13,14]. The modeling and simulation of piezoelectric materials also reveal continuous progress [15,16,17]. In piezoelectric mode, the mechanical structure transmits the targeted vertical displacement and applies stress/strain to piezoelectric materials, which then correspondingly generate electric energy and release it to external loads. When the input displacement is directly applied to a piezoelectric element without extra modulation, the large output power can only come from inputting higher displacement or using multiple piezoelectric sets [18,19,20]. Similar to the electromagnetic mode, some assisted components, e.g., lever and force amplifier, also involve enlarging the force/defamation on a piezoelectric element to promote the harvesting performance [21,22,23]. Due to their simplicity and high power density, piezoelectric materials are considered a better candidate for harvesting mechanical energy from pavements, and the generated electricity has been applied to power wireless sensor nodes, monitoring systems, and lighting signs. However, most of the reported piezoelectric schemes work under an input displacement of about 5 mm, which is still not a desired value for avoiding additional disturbance to pedestrians. Great efforts are being required to propose improved constructions to further reduce the operating displacement.

This paper, thus, develops an improved operation approach for the beam to pursue a pavement piezoelectric energy harvester (PPEH) that works under an input displacement lower than 1 mm. Different from the bending mode of conventional piezoelectric beams, the beams in the designed PPEH are axially deformed when the target displacement is loaded. A simple seesaw structure is utilized to capture the displacement and transmit it to the utilized axially deformed beams (ADBs). The basic mechanical properties of the proposed structure, with a description of the working principle, force formulization, and simulation, are described. Characterization experiments and application campaigns have been performed on the fabricated laboratory prototype. The results prove that the prototype works well in harvesting energy from a displacement lower than 1 mm, and can provide desired energy to charge a capacitor and power an environment sensing node. The rest of this paper is organized as follows: Section 2 gives the design concept and basic analyses of the proposed PPEH. Section 3 describes the fabrication of the PPEH prototype, experimental setup, and characterizations; therein, the performance comparison between ADB and conventional bending cantilever beam is conducted based on experimental results. Also, the characterization and applications of PPEH are given. Finally, the conclusions and future works are mentioned in Section 4.

## 2. Design Concept and Modeling

This paper uses a PZT (lead zirconate titanate piezoelectric ceramic) plate as a piezoelectric unit to convert mechanical energy to electricity due to its high energy conversion efficiency in d_31_ mode [24]. Based on the working principle of PZT, the mechanical stress in the PZT plate is selected as the indicator to analyze the mechanical response of ADBs, determine the configuration of each component and evaluate the electromechanical conversion performance of the energy harvester [25,26].

### 2.1. Design Concept

**Figure 1:**
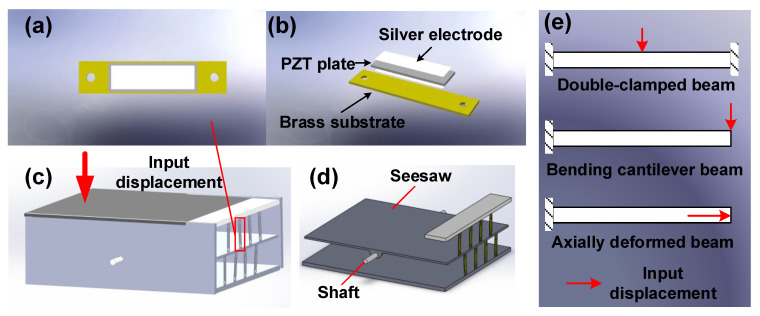
The schematic diagram for the proposed PPEH: (**a**) a single ADB with a PZT plate; (**b**) exploded view of an ADB; (**c**) the structural mode of proposed PPEH; (**d**) the structural mode of seesaw and ADBs; (**e**) the loading ways for conventional beams (**up** and **middle**) and ADB (**below**).

It is very challenging for PPEHs to efficiently harvest energy from a displacement lower than 1 mm, and the harvester based on conventional bending beams often suffers from sophisticated amplifying configurations. In this article, we proposed an ADB-based pavement piezoelectric energy harvester (Figure 1), which consists of multiple ADBs to conduct energy conversion by withstanding an axial deformation, a seesaw to capture and transmit the input displacement and necessary accessories for assembling and installation.

**Figure 2:**
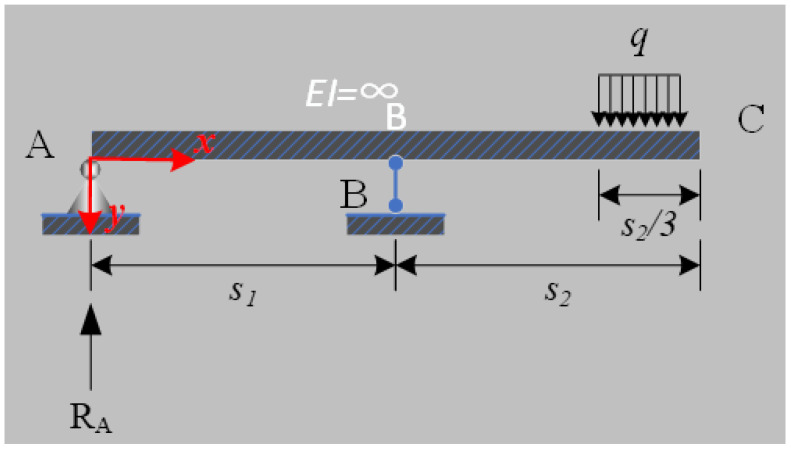
The equivalent overhanging beam model for the seesaw.

Different from the conventional bending-mode beams for piezoelectric harvesters (Figure 1e), the ADB can produce more significant and uniform mechanical stress when the same deflection is loaded. This superior capacity is the basis for the proposed PPEH to produce considerable energy under the small displacements, whose promotion effect has been validated in the development of piezoresistive accelerometers. As shown in Figure 1a,b, the PZT plate is integrated onto one of the vertical surfaces of ADB, and two through holes are placed at each end for installation. Due to the displacement restriction from ADBs on the harvesting end, the seesaw can be considered as the overhanging beam in Figure 2. The stepping excitation is regarded as a uniformly distributed load on the input end; the supporting shaft is regarded as a movable hinge; the harvesting end acts as a fixed hinge. In this model, the exciting force on ADBs is considered the reaction force on a fixed hinge, and the input displacement becomes the deflection of the input end. The detailed parametric analysis will be presented in the following subsection.

### 2.2. Modeling

In this subsection, the equivalent models for seesaw and ADBs are presented to analyze the deflection and force responses of the abovementioned components. Figure 2 shows the structural diagram for the equivalent overhanging beam under a uniform stepping load *q*. In the diagram, A is the harvesting end, B is the location of the supporting shaft, and C is the input end. The resisting arm (viz. distance between A and B) and power arm (viz. the distance between B and C) are set as *s*_1_ and *s*_2_, and *q* distributes in the first *s*_2_/3 from C. Then, the reaction force *R*_*A*_ can be calculated as

(1) $$R_{A}=\frac{5F_{p}s_{2}}{6\left(L-s_{2}\right)}$$

where *F_p_* = *qs*_2_/3 is the equivalent concentrated force of *q*. Then, the deflection curve equation of the overhanging beam is represented as

(2) $$\upsilon =\left\{\begin{array}{c} \frac{F_{p}s_{2}x}{6EI\left(L-s_{2}\right)}\left[{\left(L-s_{2}\right)}^{2}-x^{2}\right],0\leq x\leq L-s_{2}\\ -\frac{F_{p}\left(x-L+s_{2}\right)}{6EI}\left[a\left(3x-L+s_{2}\right)-{\left(x-L+s_{2}\right)}^{2}\right],L-s_{2}<x\leq L \end{array}\right.$$

where *E* and *I* are Young’s modulus of material for seesaw and structural inertia moment and *x* is the distance from point A. When *x* = *L*, the defection of input end *f*_*c*_ is

(3) $$f_{c}=-\frac{F_{p}s_{2}^{2}L}{3EI}$$

The defection in Equation (3) is also the corresponding input displacement of PPEH when the stepping pressure is loaded. It can be seen that the input displacement can be decreased by shortening the length of the seesaw (*L*) or power arm (*s*_2_). Meanwhile, this adjustment also influences the force *R*_*A*_ and may decrease the harvesting efficiency. Therefore, a more practical method is to improve the stiffness of the seesaw (e.g., promoting the elasticity modulus of the used material and optimizing the dimension). Under this circumstance, the reaction force *R*_*A*_ will trigger the ADBs to generate electric energy when a step is loaded onto the input end of the seesaw.

As for the array consisting of multiple ADBs, *R*_*A*_ is the total force acting on all beams, which each ADB withstands as tensile or pressure force. Herein, a stretched ADB below the seesaw is modeled when the force is loaded. Under the force transmitted from seesaw *F*_*A*_ (*F*_*A*_ = *R*_*A*_/N, N is the number of used ADBs), a deformation *u*_1_ and corresponding stress σ_A_ appears in ADB, which can be expressed as

(4) $$\sigma_{A}=\frac{F_{A}}{bt}=\frac{5F_{p}s_{2}}{6N\left(L-s_{2}\right)bt}$$

(5) $$u_{1}=\frac{\sigma_{A}}{E_{A}}l=\frac{5F_{p}s_{2}l}{6NE_{A}\left(L-s_{2}\right)bt}$$

where *E*_*A*_ is Young’s modulus of the ADB material, and *l*, *b*, and *t* are the length, width, and thickness of ADB, respectively. Under the similar displacement *u*_1_ at its free end, the conventional bending cantilever beam (BCB) will produce a linearly distributed stress along its length direction, and the maximum amplitude appears at the fixed end of the cantilever as

(6) $$\sigma_{B\max}=\frac{3E_{A}tu_{1}}{2l^{2}}=\frac{15F_{p}s_{2}}{12N\left(L-s_{2}\right)bl}$$

Obviously, the ADB will generate larger and more uniform stress under the same triggered displacement, and this superiority makes it much more suitable for small displacement circumstances.

**Figure 3:**
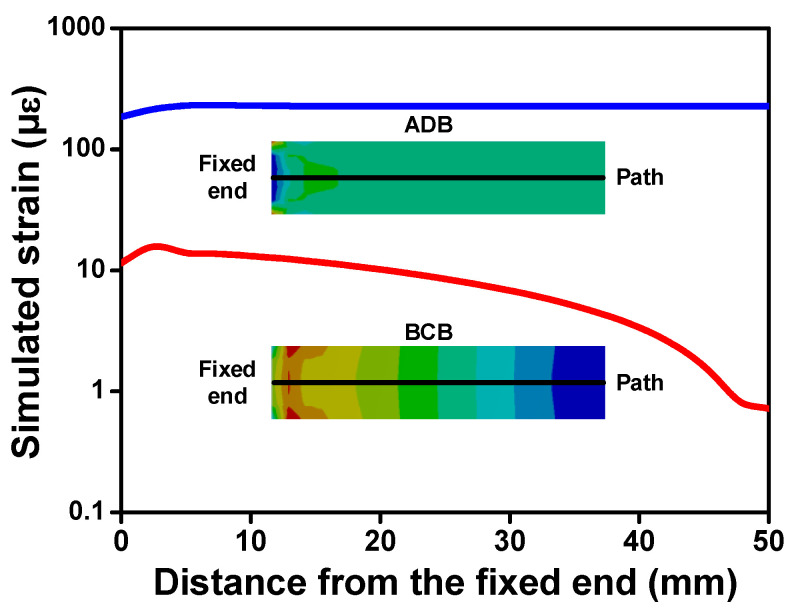
Simulated stress distributions on the top surface of ADB and BCB.

To verify the superiority of the proposed scheme, a finite element simulation is conducted by using ANSYS^®^14.5. All DOFs of their fixed end are constrained, and the same displacement of 0.5 mm is loaded onto their free ends. The displacement in ADB is along the length direction, and the force in BCB is perpendicular to the cantilever surface. The material parameters originate from brass and are included in Table 1. Figure 3 shows the simulated stress distribution on the top surface of each beam. Generally, the stress of ADB features a uniform distribution on most of the surface, except for the locally sharp changes near fixing or loading boundaries. On the contrary, the stress in BCB emerges an obvious decrease as the viewpoint is away from the fixed end.

## 3. Prototype and Experiments

The characterization experiments consist of two stages: the first stage mainly focuses on validating the advantage of ADB, in which the output performance of a single beam is tested and compared to conventional BCB; the second stage mainly focuses on the proposed PPEH, in which the laboratory prototype will be manufactured and characterized. Simple applications of the PPEH prototype are also conducted in the second stage.

### 3.1. Comparison of Single ADB and BCB

**Figure 4:**
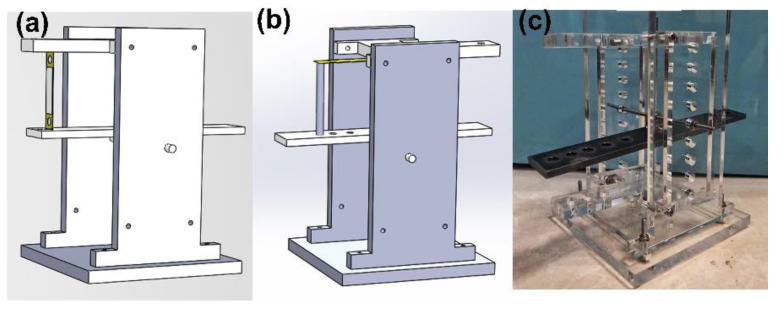
The experimental fixture for validating ADB and BCB approaches” (**a**) scheme for ADB, (**b**) scheme for BCB, and (**c**) the photo of the fabricated fixture.

In order to verify the advantage of ADB, a simple experimental fixture, as shown in Figure 4, is proposed to validate the output voltages of ADB and BCB. Herein, the same piezoelectric beams are used, but their installations differ. The beam substrate is made of a brass strip (length: 50 mm, width: 11 mm, and thickness: 0.2 mm) and a PZT plate (length: 30 mm, width: 10 mm, and thickness: 0.2 mm) centrally adheres onto the surface of each brass strip. When used as a BCB, the beam is horizontally mounted, and a pillar is placed in contact with the beam surface to transmit the external load from the seesaw mechanism. As for the ADB occasion, the beam is vertically mounted between the seesaw and fixed frame to sustain the axial displacement. An experimental device for implementing the abovementioned scheme is designed and manufactured. As shown in Figure 4c, several polymethyl methacrylate (PMMA) frames are patterned and assembled with a steel-made seesaw to transmit the input displacement to piezoelectric beams.

**Figure 5:**
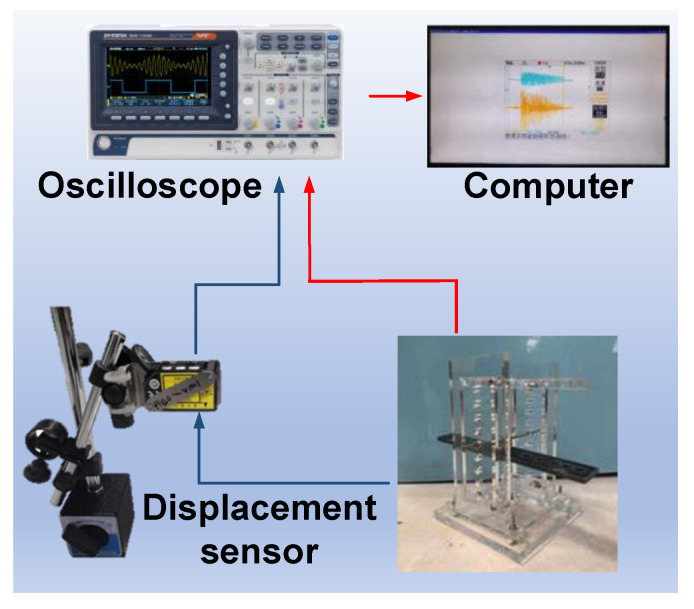
The experimental setup for beam evaluation.

**Figure 6:**
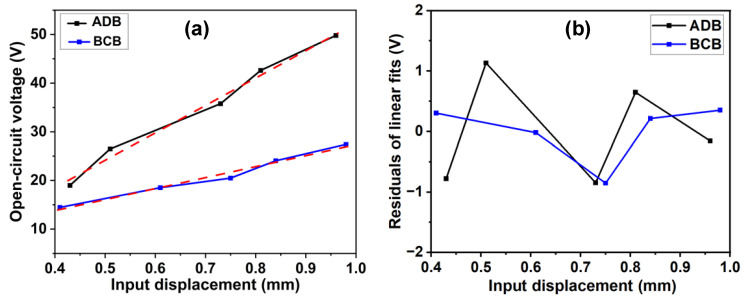
(**a**) the measured open-circuit voltage of ADB and BCB and (**b**) the residuals of linear fits.

The experimental setup for beam evaluation is illustrated in Figure 5. A laser displacement sensor (HG-C1100, Panasonic, Osaka, Japan) is utilized to measure the amplitude of input displacement, whose measuring spot is located at the input end of the seesaw. An oscilloscope (GBS1072B, GWINSTEK, Suzhou, China) is used to measure the output voltage of two piezoelectric beams under different input displacements, and the obtained results are further analyzed in a computer. The to-be-collected displacement is simulated by manual shakes. The obtained open-circuit peak-to-peak voltage for ADB and BCB under different input displacements are compared in Figure 6. The maximum tested displacement is about 0.96 mm, and a 49.8 V peak-to-peak voltage is generated by ADB. Considering the slope of each linearly fitted line, an enhancement of 356% in generated voltage is achieved by the proposed ADB scheme. Correspondingly, the measured power density also gets a maximum promotion of 1235% when a 400 kΩ load is connected to the tested PZT plates.

### 3.2. Characterizations and Applications of Proposed PPEH

**Figure 7:**
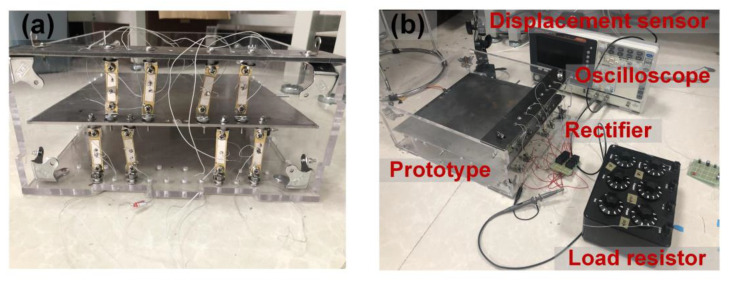
(**a**) The photo of the manufactured prototype; (**b**) the instruments for the experiment.

Figure 7 shows a laboratory prototype for the proposed PPEH, and eight ADBs are installed between the seesaw lever and fixing frame. Three PMMA boards are patterned to form the outer frames of the prototype, and steel plates are machined to form the seesaw and components for fixing ADBs. The whole size of the prototype is 35 cm × 30 cm × 15 cm (length × width × thickness), which is close to the dimension of a common brick for pavements. The experimental setup for the PPEH prototype is similar to the one for a single beam. The input excitation is simulated by the steps of a 60 kg person with a frequency of around 1 Hz. In the characterization, the PZT plates are connected in parallel, and their output voltages are rectified by a diode-bridge rectifier (KBL608) for power assessment. Then, simple applications of charging a capacitor and powering a low-consumption device are verified.

**Figure 8:**
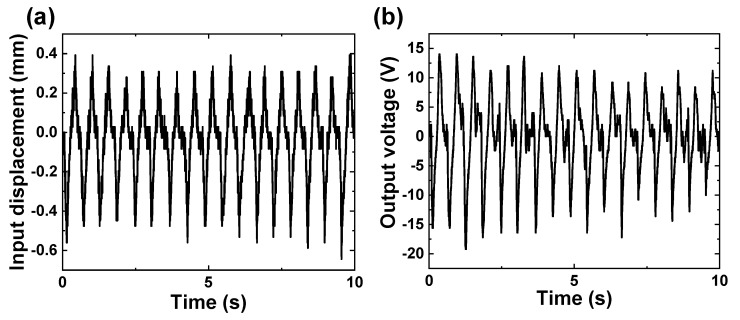
The curves of measured (**a**) input displacement and (**b**) corresponding output voltage.

**Figure 9:**
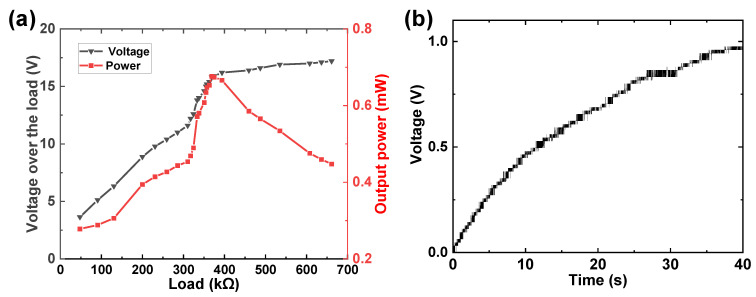
The output capacity of the proposed PPEH: (**a**) optimal load and corresponding output power; (**b**) the charged voltage over a 2200-μF capacitor.

Figure 8 shows the curves of input displacement and corresponding output voltage of one ADB in the PPEH. It can be seen that the peak-to-peak voltage of about 31.8 V is generated under the maximum displacement of around 0.6 mm, which is similar to the results of a single beam. Then, the optimal impedance and maximum output power of the proposed prototype are evaluated under a fixed input displacement of about 0.7 mm. The output current from each piezoelectric beam is rectified and then connected in parallel. Figure 9a shows the measured voltage over the varied external resistor and corresponding output power. The variation of the two curves reveals different tendencies around the optimal load, which is similar to most piezoelectric harvesters. The maximum power of 680 μW is obtained at the optimal load of 370 kΩ. This generation capacity can charge a 2200-μF capacitor up to 0.97 V in about 40 s, as shown in Figure 9b.

**Figure 10:**
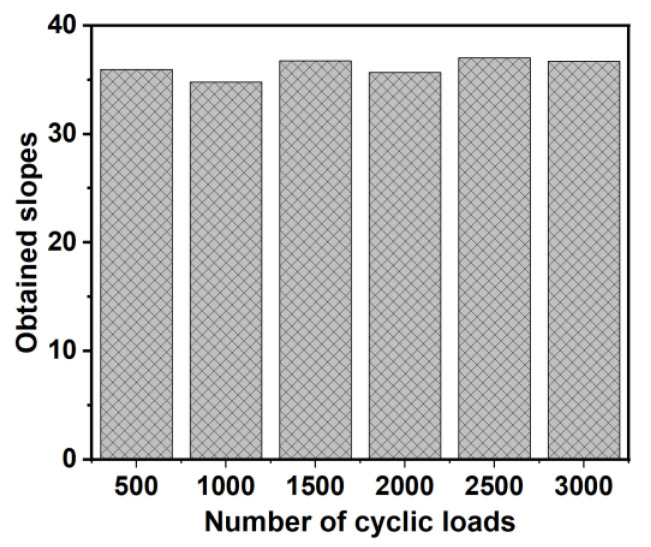
The slops of linearly fitting lines for the voltage-displacement curves after sustaining different numbers of cyclic loads.

Energy harvesting in pavements also requires outstanding durability to guarantee cost efficiency. Therefore, we conducted a simple evaluation of the performance of piezoelectric ADBs after different numbers of cyclic loads. Figure 10 shows the obtained slops of linearly fitting lines for the voltage-displacement curves after sustaining different numbers of cyclic loads. It can be seen that the relationship between the input displacement and output voltage is not very significantly influenced by the cyclic loads. Many groups have considered the service life of piezoelectric harvesting devices as 5–10 years, which is fair enough to work as a sustainable energy resource in remote areas where there is no power grid to supply energy for facilities [2,3,27].

**Figure 11:**
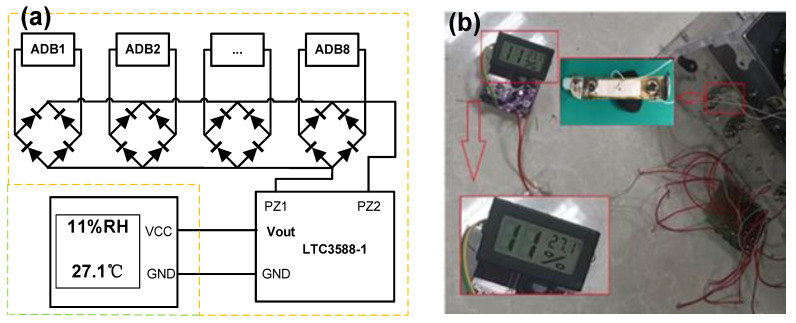
Applying proposed PPEH in powering a low-power sensing device. (**a**) Circuit diagram of the system; (**b**) Picture of the powered sensing device.

Then, a power management module (LTC3588-1) is used to adjust the voltage and power a temperature-humidity sensing device. The circuit diagram is shown in Figure 11a. The sensing device begins to work after stepping on the PPEH for about 20 s, and the state lasts about 35 s after the end of stepping (Figure 11b). Considering the real-time output ability of the proposed PPEH, the powered sensing device can work well in the periodical monitoring of environment temperature and humidity around the pavement. Moreover, with the help of an energy storage device (e.g., the abovementioned capacitor) and wireless transmitter (e.g., Bluetooth node), the device can work as a wireless sensing node to deliver the environmental parameters further.

## 4. Conclusions

In summary, the design, prototype, and characterization of a pavement piezoelectric energy harvester are investigated in this paper. With the superiority of an axially deformed piezoelectric beam in generating larger and uniform stress under a small displacement, the proposed harvester can produce favorable energy under a displacement lower than 1 mm. Compared with conventional bending cantilever beams, the utilized ADB can achieve an obvious promotion of 356% in output voltage. An experimental prototype was manufactured, and the characteristic results reveal the feasibility of using this device to harvest energy from human steps. A 2200-μF capacitor was charged, and a temperature-humidity sensing device was powered, demonstrating the possibility of powering low-consumption devices with the proposed harvester with a favorable service life. Further research on optimizing structural configuration and designing specialized interface circuits is still needed to promote harvesting efficiency. The next step also involves applying the proposed PPEH in real pavements to capture energy from human walking.

## Figures and Tables

**Table 1:** Parameters for FEA simulation.

Material Parameters			Beam Dimensions		
Young’s Modulus (GPa)	Poisson’s Ratio	Density (kg/m^3^)	Length (mm)	Width (mm)	Thickness (mm)
91	0.36	8600	50	11	0.2

## Data Availability

The data presented in this study are available upon request from the corresponding author. The data are not publicly available due to the intellectual property.
